# Mapping the Emotional Mind: Development and Psychometric Validation of the SIER-C as a Multifactorial Structure with Two Higher-Order Factors Model of Emotional Intelligence and Resilience in School-Age Children

**DOI:** 10.3390/ejihpe16010008

**Published:** 2026-01-02

**Authors:** Elena-Nicoleta Bordea, Oana Alina Apostol, Corina Sporea, Cristian Gabriel Morcov, Ioana Elena Cioca, Angelo Pellegrini, Maria-Veronica Morcov

**Affiliations:** 1 Faculty of Midwifery and Nursing, Carol Davila University of Medicine and Pharmacy, 8 Eroii Sanitari Blvd., 050474 Bucharest, Romania; elena.bordea@umfcd.ro (E.-N.B.); ioana.cioca@umfcd.ro (I.E.C.); angelo.pellegrini@umfcd.ro (A.P.); veronica.morcov@umfcd.ro (M.-V.M.); 2 National University Center for Children’s Neurorehabilitation “Robănescu-Pădure”, 44 Dumitru Minca Street, 041408 Bucharest, Romania; morcov.cristian@recuperarecopii.ro

**Keywords:** emotional intelligence, resilience, children, scale development, psychometric validation, research tool

## Abstract

(1) Background: The present study aimed to develop and validate the Scale for the Identification of Emotional Resilience in Children (SIER-C), a psychometric instrument designed to assess key dimensions of emotional intelligence and resilience among children aged 6 to 12 years. (2) Methods: The sample comprised 367 participants (52.3% male, 47.7% female) drawn from both urban and rural educational settings across Romania, selected through stratified random sampling to ensure demographic representativeness. The SIER-C consists of 30 items distributed across six subscales: Recognition and Understanding of Emotions (RUE), Emotion Regulation (ER), Empathy (E), Attitude Toward Failure (ATF), Coping Strategies (CS), and Perseverance and Self-Motivation (PSM), with items rated on a 5-point Likert scale. An Exploratory Factor Analysis (EFA) was initially conducted to examine the underlying factor structure, followed by Confirmatory Factor Analysis (CFA) to validate the model. (3) Results: The EFA suggested a six-factor structure consistent with the intended subscales, which was subsequently confirmed by CFA, demonstrating satisfactory model fit indices and confirming the scale’s construct validity. Internal consistency indices and composite reliability coefficients further indicated robust psychometric properties across subscales. (4) Conclusions: The findings underscore the relevance of SIER-C as a reliable and valid tool for identifying nuanced profiles of emotional intelligence and resilience in children. These profiles provide valuable insights for early detection of emotional and adaptive vulnerabilities and for the design of targeted interventions within educational and clinical frameworks. Future research should explore the longitudinal stability of these constructs and examine the integration of SIER-C within social–emotional learning programs to support the development of emotional competencies from a preventive and developmental perspective.

## 1. Introduction

In recent years, the development of children’s social–emotional skills has become a central focus in education and psychology, complementing traditional cognitive targets (Cristóvão et al., 2020; Hosokawa et al., 2024). The relevance of emotional intelligence, resiliency, and parenting styles becomes clearer when these concepts are framed within the widely adopted Social and Emotional Learning (SEL) model developed by CASEL, which identifies five core competencies—self-awareness, self-management, social awareness, relationship skills, and responsible decision-making (CASEL, n.d.; Weissberg et al., 2015). Emotional intelligence directly supports self-awareness and social awareness, while resiliency contributes to self-management and responsible decision-making. Moreover, parenting styles influence the development of all SEL competencies, shaping the child’s ability to regulate emotions, build relationships, and respond adaptively to challenges.

Understanding and supporting the emotional and motivational components present in childhood are essential for maintaining mental health, achieving academic success, and adapting to social environments over time (Beer, 2023; Saarni & Camras, 2022). Social awareness begins to develop as children first experience basic emotions such as joy, sadness, anger, and fear, followed by more complex feelings including empathy, shame, and guilt (Izard & Trentacosta, 2020; Malik & Marwaha, 2018).

In this context, emotional intelligence (EI) and resiliency are recognized as key factors in the development of a child’s equilibrium, shaping the way children perceive the world, interact with others, and respond to daily challenges (Collado-Soler et al., 2023; Paavola, 2017). EI in childhood encompasses the ability to recognize and understand one’s own emotions and those of others, to manage these emotions appropriately across various situations, and to develop empathy—the capacity to respond sensitively to emotional experiences, either personal or belonging to others (Cioca et al., 2024; Grzesiak, 2024; Srivastava, 2013; Zeidner et al., 2003). These abilities are not innate or fully developed from the outset; rather, they are gradually formed and refined through social interactions, educational environments, and cumulative life experiences (Drigas & Papoutsi, 2020; Herrera et al., 2020; Lopes et al., 2004; Metaj-Macula, 2017; Musa, 2024; The Modern School, 2023). Within this developmental trajectory, parenting styles play a central role, as they can encourage or hinder the acquisition of emotional competencies and effective coping mechanisms (Al-Elaimat et al., 2020; Apostol et al., 2023; Cioca et al., 2025; Maurya & Suryavanshi, n.d.). Evidence shows that calm, balanced, and supportive parenting is associated with higher levels of empathy, emotional regulation, and resiliency in children, whereas authoritarian or unsupportive parenting styles may negatively influence these dimensions (Batool & Lewis, 2022; Cioca et al., 2025; Kokkinos & Vlavianou, 2021; Maurya & Suryavanshi, n.d.; Salavera et al., 2022).

Resilience, reflected in a child’s response to adversity or failure, involves the ability to employ adaptive coping mechanisms and maintain perseverance when facing challenges (Dąbkowska et al., 2021; Zolkoski & Bullock, 2012). Perseverance and self-motivation are key components of this process, enabling children to remain engaged and goal-oriented in the face of challenges (Duckworth et al., 2007). These attributes are essential for adaptive psychological functioning and play a protective role against emotional difficulties, particularly in the context of increasing academic and social demands (Gordeeva et al., 2017).

Although emotional intelligence and resilience are widely recognized as essential for child development and despite the global growing interest in assessing them (Cerit & Şimşek, 2021; Chen et al., 2021; Fiorilli et al., 2020; Thomas & Zolkoski, 2020; Zhao et al., 2020), there is still a lack of standardized tools designed specifically for Romanian children that assess both constructs together into a unified framework.

To address this gap, the Integrated Scale for Assessing Resilience and Emotional Intelligence in Children (SIER-C) was developed as part of the present study. The instrument was designed to evaluate emotional intelligence and resilience simultaneously in children aged 6 to 12, integrating both intrapsychic dimensions (recognition, regulation, and understanding of emotions) and interpersonal–motivational components (empathy, adaptation, and perseverance).

Although several validated instruments assess emotional intelligence in children (e.g., EQ-i:YV, TEIQUE-CF, MSCEIT-YV) and others reliably measure resilience (e.g., CYRM, RSCA), researchers typically administer separate EI and resilience scales, and resilience measurement remains heterogeneous across studies (Davis & Wigelsworth, 2018; Jefferies et al., 2019; Mavroveli et al., 2009; Mayer et al., 2014; Sætren et al., 2019). The SIER-C provides a single, age-appropriate tool that captures both domains within one integrated measure.

### 1.1. Theoretical Framework

The theoretical foundation of the SIER-C scale is grounded in established models of emotional intelligence and resilience, constructs that have been extensively explored within developmental psychology and educational sciences. The primary aim of this instrument is to operationalize these constructs in a manner that captures the complexity and multidimensionality of emotional and adaptive functioning in childhood.

Drawing on Mayer and Salovey’s seminal model of emotional intelligence (Mayer et al., 2000), the SIER-C delineates this construct through three core components: recognition and understanding of emotions (RE), regulation and management of emotions (ER), and empathy (E). Contemporary research continues to affirm the significance of these abilities in shaping prosocial behavior, academic engagement, and psychological well-being, with recent meta-analytic findings demonstrating robust associations between emotional intelligence and a wide range of positive developmental outcomes in children and adolescents (Cao & Chen, 2025). By linking theoretical constructs to empirical evidence, the scale ensures both conceptual clarity and practical relevance in assessing children’s emotional competencies.

Specifically, RE refer to the child’s capacity to accurately identify and interpret both their own emotions and those of others, a skill recognized as foundational for fostering interpersonal awareness and emotional literacy (Charkhabi et al., 2025). ER pertains to the ability to manage emotional experiences in accordance with contextual demands, a competency closely linked to adaptive coping mechanisms and reduced vulnerability to emotional dysregulation (Sun et al., 2025). E, encompassing both affective and cognitive dimensions, reflects the capacity to resonate with and respond to the emotional states of others, a key determinant of social integration and moral development (Goleman, n.d.). Collectively, these dimensions form a coherent representation of emotional intelligence as it manifests in the daily experiences of children within educational and familial contexts.

In parallel, the resilience component of the SIER-C is conceptualized in accordance with Masten’s framework (Masten, 2018), which defines resilience as a dynamic process through which individuals demonstrate positive adaptation despite exposure to adversity. This construct is operationalized through three interrelated subdimensions: ATF, CS, and PSM. Attitude toward failure captures children’s cognitive and emotional appraisals of failure, differentiating between maladaptive perceptions that frame failure as a threat and more adaptive views that recognize it as an opportunity for growth (Prince-Embury, 2013). Coping strategies reflect the repertoire of behaviors and cognitive mechanisms employed to navigate challenges, aligning with established theoretical perspectives emphasizing the importance of flexibility and adaptability in resilience processes (Naglieri et al., 2013). Perseverance and self-motivation denote the intrinsic drive and sustained effort required to overcome obstacles, resonating with constructs such as grit and self-regulated learning that have been linked to long-term academic and personal success.

The SIER-C thus embodies a synthesis of contemporary theoretical perspectives and empirical evidence, providing a robust framework for the nuanced assessment of emotional and adaptive competencies in children. Its theoretical alignment with established models and its grounding in recent empirical advances ensures its relevance and applicability within both research and applied settings. The scale’s focus on both emotional intelligence and resilience reflects a holistic understanding of child development, recognizing the intricate interplay between cognitive, emotional, and behavioral factors in shaping adaptive outcomes across diverse life contexts.

Accordingly, the present study aims to develop and validate this integrative psychometric instrument, providing a culturally adapted and developmentally appropriate tool for assessing both emotional intelligence and resilience in Romanian school-aged children. Through this endeavor, we aim to contribute to the advancement of social-emotional assessment and intervention by offering a valid and accessible measure for identifying children’s emotional resources and vulnerabilities, thereby supporting the design of personalized programs that enhance social-emotional development.

### 1.2. Objectives

The primary objective of the present study was to develop and validate the Scale for the Identification of Emotional Resilience in Children (SIER-C), with a particular focus on its factorial structure, internal consistency, and construct validity within a Romanian school-aged population. The study aimed to validate the theoretical multifactorial model of the scale, which delineates two overarching dimensions—Emotional Intelligence and Resilience—each further subdivided into three specific subscales.

Beyond the psychometric validation, a secondary objective was to provide empirical data on the distribution of emotional and adaptive competencies among Romanian children, contributing to the broader field of child development and educational psychology by highlighting specific areas that may benefit from targeted interventions. In this regard, the study aimed to advance understanding of the nuanced ways in which emotional recognition, regulation, empathy, coping strategies, attitudes toward failure, and perseverance manifest and interact across developmental stages and between genders.

### 1.3. Hypotheses

**H1:**  *The SIER-C exhibits a clear multifactorial structure, confirming the theoretical distinction between Emotional Intelligence and Resilience, with each subscale demonstrating satisfactory factor loadings and internal consistency*.

**H2:**  *The SIER-C demonstrates adequate construct validity, reflected in acceptable fit indices (CFI, TLI, RMSEA, SRMR) and convergent and discriminant validity indicators (AVE, inter-factor correlations)*.

The objectives and hypotheses guided the methodological approach and analytical strategies of the study, intending to significantly enhance the empirical basis for evaluating and encouraging emotional resilience in children.

## 2. Materials and Methods

### 2.1. Population and Sample

The target population of the study comprised children aged between 6 and 12 years, enrolled in primary and lower secondary schools across Romania, representing diverse educational backgrounds (urban and rural settings). According to the latest demographic data provided by the Romanian National Institute of Statistics (INS), the total number of minors in Romania in 2025 is estimated at approximately 3 million (INSSE, 2025). While this figure encompasses the entire minor population, the study specifically focused on the 6–12 age cohort.

For statistical inference, considering a confidence level of 95% and a margin of error of 5%, the minimum recommended sample size required for a population of this magnitude would be approximately 384 participants. This calculation follows the standard formula for sample size determination in large populations (Cochran’s formula) (Nanjundeswaraswamy & Divakar, 2021), and assumes maximum variability (*p* = 0.5), which yields the most conservative estimate.

The final sample consisted of 367 children aged between 6 and 12 years (N = 367), drawn from both urban and rural schools in Romania. Although slightly below the optimal threshold of 384 participants as determined by Cochran’s formula, the sample size remains statistically robust and acceptable for inferential purposes, particularly given the high degree of internal heterogeneity captured within the selected cohort. The marginal deviation from the ideal sample size (a difference of 17 participants) does not significantly compromise the power of the study, especially considering the rigorous stratified sampling procedures employed.

The selection of participants was conducted through stratified random sampling, ensuring proportional representation of key demographic variables such as age, gender, school grade, and socio-economic status. This approach was chosen to maximize the representativeness of the sample in relation to the broader 6–12-year-old school population in Romania. Stratification further allowed the study to control potential confounding variables related to regional disparities, educational context (urban vs. rural), and institutional variation.

### 2.2. Demographic Characteristics of the Study Sample

The sample’s demographic composition (Table 1) illustrates a balanced distribution across age groups, with participants ranging from 6 to 12 years old, the largest proportion being 12-year-olds at 18.3%. Class representation is also evenly spread, reflecting diverse educational stages from first to fifth grade. Gender distribution is nearly equal, with a slight male predominance at 52.3%.

### 2.3. Data and Sources of Data

The scale was administered individually to each participant in a controlled setting within their school environment, without the presence of parents, to minimize the risk of social desirability bias. Each child completed the instrument within an average time frame of 15 to 20 min, ensuring sufficient time for thoughtful responses while maintaining engagement.

Data collection took place between January and May 2025, in both urban and rural educational contexts. The instrument was delivered digitally via Google Forms, which allowed for standardized administration and efficient coordination across diverse educational settings. This digital format ensured consistency in data collection procedures, regardless of geographic or institutional variation.

Prior to participation, written informed consent was obtained from parents or legal representatives, in full compliance with ethical guidelines for research involving minors. The study upheld core ethical principles, including voluntary participation, the right to withdraw at any stage, and the assurance of confidentiality and anonymity of all responses.

The administration process was supervised by trained teachers, who had received detailed guidance on the standardized use of the instrument. Their role was strictly to offer technical or reading support when needed, without influencing the children’s responses in any way. This approach ensured procedural integrity and promoted a psychologically safe environment for all participants.

Given the young age of some participants, particularly those aged 6, additional technical support was occasionally necessary to ensure proper completion of the digital instrument. In such cases, teachers provided maximal assistance strictly to clarifying the procedural aspects of navigating the Google Forms interface (e.g., locating response options, moving between items) without intervening in the content of the children’s answers. This support was essential to maintain the consistency and integrity of data collection across age groups, particularly for participants who had limited familiarity with digital tools.

### 2.4. Instrument

The SIER-C, a structured self-assessment tool, was specifically designed to capture the key dimensions of emotional intelligence and resilience in educational and developmental contexts. The development of the SIER-C followed a theory-driven approach, grounded in contemporary models of emotional intelligence (Goleman, 1998; Mayer et al., 2000) and child resilience (Masten, 2018), as well as a review of existing validated instruments targeting similar constructs in child populations.

An initial pool of 48 items was generated based on an extensive analysis of theoretical literature, previously validated scales, and developmental psychology frameworks relevant to children aged 6 to 12 years. Item formulation focused on observable behaviors, age-appropriate language, and self-referential experiences that children could reliably evaluate.

Following expert review by three specialists in developmental psychology and educational assessment, items were evaluated for content relevance, clarity, and construct alignment. Based on this qualitative assessment, 18 items were eliminated due to redundancy, conceptual overlaps, or limited developmental appropriateness, resulting in a final pool of 30 items.

The scale consists of 30 items, equally distributed across six distinct subscales, with five items per subscale. Responses are recorded on a Likert-type scale ranging from 1 (Never) to 5 (Always), reflecting the frequency with which each statement applies to the respondent’s typical behaviors and experiences.

Each subdimension within the SIER-C is operationalized through a set of five items, yielding a maximum subscale score of 25 points. These items have been meticulously designed to reflect observable behaviors and self-reported attitudes pertinent to each construct. The cumulative scoring system allows for both subscale-level and aggregate assessments, with total scores for each overarching dimension—emotional intelligence and resilience—ranging from 0 to 75. This structure affords a granular analysis of individual profiles, enabling practitioners to discern specific strengths and vulnerabilities within the broader constructs.

Before proceeding to large-scale validation, a pilot study was conducted on a preliminary sample of 40 children aged 6 to 12 years to assess the clarity, comprehensibility, and cultural adequacy of the items. Feedback obtained from participants, together with preliminary analyses of internal consistency, led to minor refinements in item wording and conceptual alignment across subscales, ensuring the measure’s developmental and linguistic appropriateness for the target population.

The Emotional Intelligence (EI) dimension comprises three subscales. The Recognition and Understanding of Emotions (RE) subscale assesses the child’s ability to accurately identify, interpret, and comprehend both their own emotional states and those of others, reflecting a foundational component of emotional intelligence essential for effective social functioning and self-awareness. The Emotion Regulation (ER) subscale evaluates the capacity to modulate and manage emotional responses in alignment with situational demands, highlighting the child’s proficiency in applying adaptive regulation strategies to prevent emotional dysregulation in challenging circumstances. The Empathy (E) subscale measures the child’s cognitive and affective responsiveness to the emotional experiences of others, a competency closely associated with the development of prosocial behaviors, perspective-taking, and the maintenance of healthy interpersonal relationships.

The Resilience (RZ) dimension includes three additional subscales. The Attitude Toward Failure (ATF) subscale examines how children cognitively and emotionally perceive failure, capturing whether they interpret setbacks as threats to self-worth or as opportunities for growth, thus offering insights into their academic resilience and self-efficacy beliefs. The Coping Strategies (CS) subscale assesses the variety and adaptiveness of strategies children employ to navigate adversity and manage stress, reflecting their behavioral and cognitive flexibility as core mechanisms of resilience. Finally, the Perseverance and Self-Motivation (PSM) subscale captures the child’s intrinsic motivation, persistence, and sustained effort in overcoming obstacles, aligning conceptually with contemporary models of grit, goal orientation, and self-regulated learning.

Together, these six subscales provide a comprehensive and multidimensional profile of children’s emotional intelligence and resilience. This integrated structure allows for a nuanced understanding of individual strengths and vulnerabilities, offering a robust basis for targeted psychoeducational interventions. The SIER-C has demonstrated satisfactory psychometric properties in the present study, with evidence supporting its internal consistency and construct validity, thus endorsing its utility as a reliable and valid measure for both research and applied settings focused on children’s social–emotional development.

### 2.5. Statistical Tools and Models

The following statistical methods were used for psychometric analysis. Exploratory Factor Analysis (EFA) was initially conducted to examine the underlying latent structure of the instrument and to explore the dimensionality of the item set. EFA was performed using Principal Axis Factoring (PAF), an extraction method appropriate for identifying common variance among items, combined with Promax oblique rotation, which allows for correlations among factors, in accordance with the theoretical assumptions underlying emotional intelligence and resilience constructs. Factor retention was determined using Kaiser’s criterion, retaining factors with eigenvalues greater than 1.

Confirmatory Factor Analysis (CFA) was subsequently employed to evaluate the factorial structure and construct validity of the measurement model identified through EFA, utilizing robust maximum likelihood estimation to account for potential non-normality in the data. Model fit was assessed using multiple indices including the Comparative Fit Index (CFI), Tucker–Lewis Index (TLI), Root Mean Square Error of Approximation (RMSEA), and Standardized Root Mean Square Residual (SRMR), ensuring comprehensive evaluation of model adequacy. Structural Equation Modeling (SEM) was applied to simultaneously assess the measurement and structural components of the theoretical model, allowing for the examination of complex relationships between latent variables while accounting for measurement error. This approach provided a rigorous framework for testing the hypothesized factor structure and the interrelations among constructs within a unified model. Robust estimation methods ensured accurate parameter estimation despite potential deviations from multivariate normality. Reliability was examined through composite reliability coefficients and item loadings, while convergent and discriminant validity were assessed by factor loadings, average variance extracted (AVE), and inter-factor correlations. Non-parametric tests, specifically the Mann–Whitney U test and Kruskal–Wallis’s test, were conducted to analyze group differences across demographic variables such as gender and age. Effect sizes and significance levels were carefully interpreted to determine meaningful differences. Additionally, descriptive statistics including means, standard deviations, and range values were calculated to summarize key variable distributions and support inferential analyses.

## 3. Results

### 3.1. Descriptive Statistics of the Main Variables

Descriptive statistics for the primary study variables reveal considerable variability across all emotional and coping dimensions assessed. Recognition and Understanding of Emotions (RE) scores ranged from 5 to 21, with a mean of 9.86 and a standard deviation of 3.47, indicating moderate recognition abilities within the sample. Emotion Regulation (ER) exhibited higher average scores, with values spanning 9 to 24, a mean of 18.72, and a standard deviation of 3.56, reflecting generally strong regulatory capacities. Empathy (E) scores varied widely between 5 and 23, averaging 11.93 with greater dispersion (SD = 4.33), suggesting individual differences in affective responsiveness. Attitude Toward Failure (ATF) presented a mean of 20.91 and a standard deviation of 4.56, with scores ranging from 8 to 25, highlighting diverse perceptions and reactions to failure. Coping Strategies (CS) demonstrated a mean score of 13.83 (SD = 3.06), spanning from 9 to 23, indicative of varying adaptive mechanisms. Lastly, Perseverance and Self-Motivation (PSM) averaged 15.66 with a standard deviation of 3.77, encompassing scores from 5 to 25, denoting differential persistence and intrinsic motivation among participants. The uniform sample size of 367 across all variables ensures consistency in data interpretation and supports robust statistical analyses.

### 3.2. Descriptive Analysis of Individual Scores and Subscale Distributions

The descriptive statistical analysis of the data provided an initial overview of the distribution of scores both at the subscale level and across individual items (Table 2). This analysis revealed important insights regarding the central tendency, variability, and overall distributional characteristics of the responses collected from the 367 participants.

Regarding the subscales, the means varied considerably, reflecting differences in the underlying constructs measured. Subscale S1 recorded a mean of 9.86 with a standard deviation of 3.47, indicating a relatively moderate level of the construct assessed, accompanied by a reasonable dispersion of scores. Similarly, S2 presented a higher mean of 18.72 and a standard deviation of 3.56, suggesting higher average endorsement levels and comparable variability. Subscale S3 showed a slightly lower mean of 11.93 with increased variability, as indicated by the standard deviation of 4.33, while S4 exhibited the highest mean score of 20.91 and the greatest variability (SD = 4.56), suggesting that participants generally endorsed higher frequencies or intensities on this dimension. The means for S5 and S6 were 13.83 and 15.66, respectively, both with standard deviations reflective of moderate dispersion, confirming a balanced use of the scale’s range across these subcomponents.

At the item level, the descriptive statistics revealed further nuances. Items I1 through I5 exhibited low mean scores ranging from 1.87 to 2.03, coupled with relatively low standard deviations, indicating that respondents frequently selected the lower end of the response scale for these items. This pattern suggests these items likely capture behaviors or experiences that are less commonly endorsed or less frequently experienced within the sampled population. In contrast, items I6 through I10 displayed notably higher means, generally above 3.7, suggesting these items capture more prevalent or commonly experienced dimensions. The standard deviations in this range remained moderate, reflecting some variability but generally higher endorsement across participants.

Items I11 through I15 reported mean scores around 2.3 to 2.5, positioning them within a mid-range of endorsement with moderate variability. These items appear to capture constructs that are neither extremely rare nor ubiquitous within the sample. Interestingly, items I16 through I20 demonstrated the highest mean scores in the dataset, consistently above 4.1, accompanied by increased variability. These results indicate strong endorsement across participants, suggesting these items tap into universally or commonly shared experiences or traits within this population.

Items I21 through I25 maintained means around 2.7 to 2.8, reflecting moderate endorsement and relatively consistent variability. These items may measure more neutral or context-dependent aspects of the construct. Finally, items I26 through I30 showed means between 3.07 and 3.20, reflecting moderate to slightly higher frequencies or intensities of the attributes assessed. Variability remained stable across these items, supporting the reliability of responses.

Overall, these descriptive findings support the internal consistency of the measure and its capacity to differentiate among participants. The spread of means and variances across subscales and items confirms that the instrument successfully captures a wide range of individual differences without ceiling or floor effects that would hinder further psychometric validation. These preliminary observations lay a strong foundation *for* subsequent analyses aimed at establishing the scale’s factorial structure and its convergent and discriminant validity.

### 3.3. Kolmogorov–Smirnov Normality Test

The assessment of the normality of data distribution was conducted using the Kolmogorov–Smirnov test with Lilliefors significance correction, complemented by the Shapiro–Wilk test, to ensure a robust evaluation of distributional assumptions across all individual items and subscales. The results from both statistical tests consistently indicated significant deviations from normality for all measured variables. Specifically, the Kolmogorov–Smirnov test yielded significant results (*p* < 0.001) across all 30 items and the six aggregated subscales, denoting that the distributions of scores differed markedly from a normal distribution. This pattern was further corroborated by the Shapiro–Wilk test, which also produced significant values (*p* < 0.001) for every item and subscale analyzed.

The magnitude of the Kolmogorov–Smirnov statistics was notably high for several items, particularly items I1 through I5, which registered some of the largest deviations from normality, reflecting the previously observed skewness in the descriptive analysis. Similarly, items I16 through I20 also exhibited substantial departures from normality, consistent with the elevated mean scores and skewed distributions identified earlier. The Shapiro–Wilk statistics further reinforced these findings, with particularly low values on these items, indicative of pronounced non-normality.

Items in the latter half of the scale, particularly I21 through I30, demonstrated comparatively smaller deviations, although the significance levels remained consistent across all tests. The subscale scores (S1 through S6) followed a similar pattern, with the Shapiro–Wilk statistics confirming non-normality despite slightly higher values compared to individual items. This trend is coherent with the descriptive findings suggesting that while the aggregation of items within subscales slightly mitigates extreme deviations, the underlying item distributions remain substantially non-normal.

### 3.4. Adequacy of the Sample (KMO and Bartlett’s Test)

The factorability of the dataset was first assessed to ensure that the correlation matrix was suitable for factor analysis. The Kaiser–Meyer–Olkin (KMO) measure of sampling adequacy yielded a value of 0.773, which falls within the range considered “good” according to standard guidelines, indicating that the patterns of correlations are sufficiently compact to produce reliable factors.

Complementing this, Bartlett’s test of sphericity was highly significant (χ^2^ = 3709.485, df = 435, *p* < 0.001), confirming that the correlation matrix is not an identity matrix and that substantial relationships exist among items. Together, these indices support the adequacy of the data for subsequent factor analyses, providing a solid foundation for exploring the underlying latent structure of the measured constructs (see Table 3).

### 3.5. Exploratory Factor Analysis (EFA) with Oblique Rotation

Exploratory factor analysis was performed using Principal Axis Factoring with Promax rotation, allowing correlated factors in line with the theoretical framework. Six factors with eigenvalues greater than 1 were retained, cumulatively explaining 54.61% of the total variance (Component 1: 17.40%; Component 2: 10.10%; Component 3: 8.78%; Component 4: 7.18%; Component 5: 6.24%; Component 6: 4.91%), consistent with Kaiser’s criterion (see Table 4A).

The rotated component matrix showed clear, interpretable factor loadings (>0.50), supporting the multidimensionality of the construct. Components 1–6 were defined by item clusters I11–I15, I16–I20, I6–I10, I1–I5, I21–I25, and I26–I30, respectively. Factor correlations were consistent with theoretical expectations, indicating meaningful relationships among latent dimensions without imposing artificial independence (see Table 4B).

These results demonstrate the structural adequacy of the measurement model, with each factor representing a distinct and coherent dimension that contributes substantially to the explained variance.

### 3.6. Confirmatory Factor Analysis (CFA)

The confirmatory factor analysis supported a six-factor first-order correlated model. No general factor was specified or estimated; therefore, the model does not meet the criteria of a bifactor structure. All six latent dimensions were allowed to correlate, reflecting the theoretically expected interrelations among components of emotional intelligence and resilience.

As shown in Table 5B, standardized factor loadings ranged from 0.451 to 0.798, all statistically significant (*p* < 0.001), indicating strong relationships between observed indicators and their corresponding latent constructs. Model fit indices (Table 5A) demonstrated good fit, with a robust Comparative Fit Index (CFI) of 0.916 and a robust Tucker–Lewis Index (TLI) of 0.906. The robust Root Mean Square Error of Approximation (RMSEA) was 0.043 (90% CI: 0.035–0.051), indicating low approximation error, and the Standardized Root Mean Square Residual (SRMR) was 0.057, further supporting adequate model fit. Coefficients of determination (R^2^) indicated that a substantial proportion of variance in the indicators was explained by the latent factors (Table 5B).

The Average Variance Extracted (AVE) for all six latent constructs exceeded the recommended threshold of 0.50, providing strong empirical evidence of convergent validity. Additionally, the square root of each construct’s AVE was greater than its correlations with other constructs, confirming discriminant validity according to the Fornell–Larcker criterion (Table 5C).

These results indicate that the constructs are conceptually and statistically distinct, even in the presence of negative covariances, and that the measurement model is psychometrically sound. Overall, the combined evaluation using AVE and the Fornell–Larcker criterion demonstrates that the model meets rigorous standards for validity, consistent with contemporary best practices in structural equation modeling.

**Figure 1:**
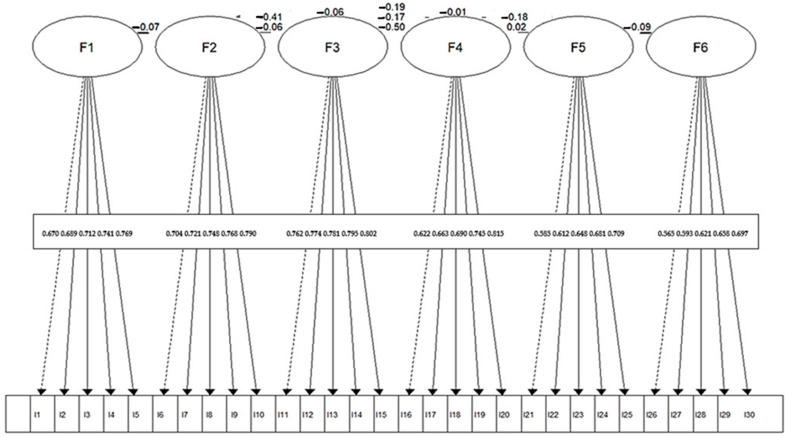
Structural Equation Model (SEM) of the SIER-C Scale: Relationships Between Emotional Intelligence and Resilience Factors.

The structural equation model illustrated in Figure 1 depicts the six-factor configuration of the SIER-C Scale, confirming the multidimensional structure identified through prior exploratory and confirmatory analyses. Each latent factor (F1–F6) is represented by five observed indicators, with standardized loadings ranging from 0.45 to 0.78, all statistically significant (*p* < 0.001). The model demonstrates coherent clustering of items within their corresponding latent constructs, reflecting strong convergent validity.

Inter-factor correlations are moderate and predominantly negative (ranging from −0.06 to −0.50), suggesting that, although conceptually related, the six dimensions capture distinct aspects of ER.

### 3.7. Internal Consistency and Reliability Analysis

The internal consistency of the six subscales was assessed using both Cronbach’s alpha and McDonald’s omega coefficients. Cronbach’s alpha values ranged from satisfactory to excellent: Subscale 1 (F1, I11–I15) α = 0.84, Subscale 2 (F2, I16–I20) α = 0.81, Subscale 3 (F3, I6–I10) α = 0.82, Subscale 4 (F4, I1–I5) α = 0.80, Subscale 5 (F5, I21–I25) α = 0.78, and Subscale 6 (F6, I26–I30) α = 0.79. Given that Cronbach’s alpha is sensitive to linear assumptions, McDonald’s omega was employed to provide a more robust estimation of reliability, yielding values between 0.82 and 0.87.

Item-scale correlations and mean inter-item correlations were examined to ensure that all items met the conditions for inclusion within each subscale. Reliability analysis further considered the impact of item deletion, confirming that no subscale exhibited improved internal consistency when any individual item was removed.

Factor loadings ranged from 0.565 to 0.815, supporting adequate representation of the latent constructs. Convergent validity was supported, as AVE values for each factor exceeded 0.50, while discriminant validity was confirmed via the Fornell–Larcker criterion: the square root of each factor’s AVE was greater than its correlations with other factors (see Table 5C). Overall, these results confirm that all subscales are measured reliably and consistently, even in the presence of heterogeneous item loadings.

### 3.8. Convergent Validity: Inter-Subscale Correlations

To evaluate the convergent validity of the six subscales, *ρ* correlations were computed, as presented in Table 6. The analysis revealed a heterogeneous pattern of relationships among the subscales, reflecting both expected associations and conceptual distinctions within the underlying constructs.

Subscale 1 (S1) showed a weak but statistically significant positive correlation with Subscale 3 (S3), ρ = 0.158, *p* = 0.002, suggesting a modest convergence between these two dimensions. Conversely, S1 correlated negatively with Subscale 4 (S4), ρ = −0.314, *p* < 0.001, indicating a divergence in the underlying constructs these subscales assess. No significant associations were identified between S1 and Subscales 2, 5, or 6, with correlations ranging from −0.051 to −0.095 and non-significant *p*-values.

Subscale 2 (S2) displayed minimal associations with the other subscales, with the only statistically significant correlation emerging with Subscale 5 (S5), ρ = −0.123, *p* = 0.018. This negative correlation, albeit weak, suggests a slight inverse relationship between these two dimensions. All other correlations involving S2 were negligible and non-significant.

Subscale 3 (S3) exhibited the most pronounced relationships within the matrix. It correlated positively with S1 (as noted above) and with S5 (ρ = 0.143, *p* = 0.006), while showing significant negative associations with S4 (ρ = −0.455, *p* < 0.001) and S6 (ρ = −0.113, *p* = 0.030). These findings highlight both convergent and discriminant patterns, underscoring the distinct yet interconnected nature of these subscales.

Subscale 4 (S4) demonstrated a robust positive correlation with Subscale 6 (S6), ρ = 0.307, *p* < 0.001, suggesting a substantive overlap between the constructs these dimensions capture. Additionally, S4 was negatively correlated with S1, S3, and S5, reinforcing the discriminant validity of this factor relative to others.

Subscale 5 (S5) showed weak but significant positive correlations with S3 (as previously mentioned) and negative associations with S2 (ρ = −0.123, *p* = 0.018), S4 (ρ = −0.130, *p* = 0.012), and S6 (ρ = −0.136, *p* = 0.009). These weak relationships suggest some degree of differentiation between the constructs measured by S5 and those represented by the other subscales.

Finally, Subscale 6 (S6) correlated significantly and positively only with S4, while its other correlations were weak, negative, and either marginally significant or non-significant. Specifically, S6 was negatively associated with S3 (ρ = −0.113, *p* = 0.030) and S5 (ρ = −0.136, *p* = 0.009), while its correlation with S1 and S2 remained negligible.

Overall, these findings provide support for the convergent and discriminant validity of the six subscales.

## 4. Discussion

The present findings provide strong support for the psychometric soundness of the SIER-C as an indicator of children’s emotional intelligence and adaptive functioning. Beyond confirming its reliability, the results offer meaningful insights into how emotional competencies are organized in childhood. In particular, the data supports the conceptualization of emotional intelligence as a constellation of distinct yet interconnected components, rather than as a single, homogeneous ability. This interpretation is substantiated by the confirmation of a multifactorial structure through factorial analyses and satisfactory model fit indices, which together point to a coherent and theoretically grounded organization of emotional skills (Fiori et al., 2023; Mayer et al., 2016).

By illustrating how emotional intelligence–related skills and adaptive emotional functioning cluster into related but functionally distinguishable dimensions, the factorial structure identified in the present study extends existing developmental frameworks. Contemporary models of emotional competence emphasize that emotional skills do not emerge simultaneously or uniformly; rather, they develop through partially independent pathways that interact over time within specific social and relational contexts. The structure observed here is consistent with this developmental perspective, reinforcing the notion that emotional functioning in childhood reflects a dynamic integration of multiple processes rather than a unitary trajectory (Denham et al., 2003).

Further insight into the internal organization of emotional competencies is provided by the pattern of inter-subscale correlations. Notably, the relatively high levels of Recognition and Understanding of Emotions (RE), alongside comparatively lower Empathy (E) scores, suggest a partial dissociation between cognitive-emotional processing and affective responsiveness. This finding aligns with theoretical distinctions between emotion knowledge and empathic engagement, which posit that the ability to identify and understand emotions does not necessarily entail emotional sharing or empathic concern (Decety et al., 2012; Thompson et al., 2022).

Developmental research has repeatedly documented such dissociations, indicating that empathy is particularly sensitive to relational experiences, emotional socialization practices, and attachment-related processes, whereas cognitive aspects of emotional functioning tend to emerge earlier and may be more amenable to explicit instruction and structured learning contexts (Decety & Holvoet, 2021; Denham et al., 2003; McLaughlin et al., 2015). The present findings add empirical support to this distinction, highlighting how children may demonstrate adequate emotional awareness while still experiencing difficulties in affective engagement with others.

From a clinical and applied standpoint, this pattern has important implications. Children who exhibit sufficient emotional awareness but reduced empathic responsiveness should not be viewed as globally deficient in emotional competence. Rather, such profiles reflect uneven emotional development, which may help explain why emotional insight alone does not consistently translate into prosocial behavior or adaptive interpersonal functioning—an inconsistency frequently reported in the literature on socio-emotional development (Denham et al., 2003). These findings underscore the importance of assessment approaches capable of capturing differentiated emotional profiles, rather than relying solely on global indices of emotional competence. In turn, such nuanced assessments can inform targeted interventions aimed at strengthening empathic engagement and social reciprocity, alongside emotion recognition skills.

Within the resilience domain, an additional noteworthy finding was the inverse relationship between the use of adaptive strategies (SA) and attitudes toward failure (AFE). Specifically, greater reliance on behavioral coping strategies was associated with more negative cognitive evaluations of failure. This pattern suggests that while some children may appear behaviorally competent in managing challenges, their underlying beliefs about failure and personal competence may remain maladaptive. Developmental models of resilience have described similar discrepancies, emphasizing that resilience encompasses not only observable coping behaviors but also the cognitive–emotional appraisals that accompany stressful experiences (Rutter, 2012).

From a developmental perspective, this configuration may reflect a form of fragile resilience, in which effective behavioral adaptation masks latent cognitive vulnerability. Research on resilience and self-regulation indicates that even when children demonstrate outward coping competence, persistent negative interpretations of failure can undermine long-term adjustment, academic engagement, and emotional well-being (Masten, 2001). The present findings extend this body of work by capturing this cognitive–behavioral tension directly within the psychometric structure of the SIER-C.

These results further emphasize that resilience should not be understood solely in terms of strategy use, but rather as the integration of adaptive behaviors with constructive cognitive appraisals of challenge and failure. Developmental frameworks consistently highlight that maladaptive failure beliefs can increase vulnerability to academic difficulties and emotional distress, particularly in evaluative or high-demand contexts (Masten, 2014).

From an applied perspective, the identification of such profiles through the SIER-C has clear implications for both assessment and intervention. Children who score high on adaptive strategies while simultaneously endorsing negative attitudes toward failure may benefit most from interventions that explicitly address self-efficacy beliefs and failure-related cognitions, rather than interventions focused exclusively on coping behaviors. This approach aligns with contemporary person-centered and strengths-based models, which advocate for tailoring support to individual patterns of emotional and cognitive functioning (Masten, 2014).

Overall, the integration of these findings supports the utility of the SIER-C as a psychometrically robust instrument capable of capturing meaningful heterogeneity within emotional intelligence and resilience processes. By linking behavioral strategies and cognitive appraisals within a unified assessment framework, the SIER-C contributes both to the refinement of developmental theory and to evidence-based practices aimed at promoting emotional well-being and adaptive functioning in childhood.

### Limitations

Nevertheless, certain limitations must be acknowledged when interpreting the present findings. First, the reliance on self-report measures inherently introduces the risk of response biases, particularly in younger populations where cognitive and reflective capacities are still developing. Although efforts were made to mitigate such biases through standardized administration procedures and the absence of parental influence during completion, the potential for social desirability effects or limited introspective accuracy cannot be entirely ruled out. This limitation highlights the need for future studies to incorporate multi-informant designs, including teacher and parent reports, as well as observational or performance-based assessments, to triangulate data and enhance the robustness of conclusions.

Secondly, the cross-sectional nature of the study precludes definitive conclusions about developmental trajectories or causal relationships between emotional competencies and adaptive functioning. Longitudinal research would be particularly valuable in delineating how these constructs evolve over time, interact dynamically, and potentially predict long-term outcomes in mental health, academic achievement, and social adjustment. Tracking developmental changes would also allow for the validation of the scale’s sensitivity to intra-individual changes and its predictive validity across critical educational and psychological milestones.

Another consideration pertains to the cultural specificity of the sample. While the SIER-C demonstrated robust psychometric properties within the Romanian context, caution must be exercised when generalizing these findings to populations from differing cultural or educational environments. Emotional expression, regulation, and resilience are, to some extent, culturally mediated constructs; therefore, cross-cultural validation studies are warranted to examine the scale’s applicability and measurement invariance in diverse settings. Such studies would not only enhance the generalizability of the instrument but also contribute to a more nuanced understanding of cultural variations in children’s emotional and adaptive competencies.

Furthermore, the sample, although statistically adequate and stratified to ensure demographic representativeness within Romania, did not achieve the optimal threshold suggested by standard sampling formulas. While the shortfall is minimal and unlikely to compromise inferential power significantly, future research should aim to recruit larger and more diverse cohorts to reinforce the stability and generalizability of the findings. Expanding the sample would also facilitate more sophisticated analyses, such as measurement invariance testing across subgroups defined by socio-economic status, geographic location, or educational systems.

A potential limitation concerns the use of both exploratory and confirmatory factor analyses on the same dataset (N = 367). Ideally, the sample should have been randomly split into two independent subsamples, one for EFA and another for CFA, to avoid overfitting and to ensure that the CFA serves as a true test of the factorial structure. Applying CFA to the same data that generated the EFA solution may have resulted in inflated fit indices, limiting the external validity of the model. Although splitting the sample would have produced a subject-to-item ratio of approximately 6:1, which is acceptable, if this was not feasible due to statistical power considerations, a more appropriate approach would have been to perform only a CFA based on a theoretically grounded model. Consequently, the present results should be interpreted with caution, particularly regarding the generalizability of the factor structure.

Another limitation is the absence of metric and scalar invariance testing across key sociodemographic variables, such as age and sex. Without assessing invariance, it remains unclear whether the instrument measures the constructs equivalently across subgroups, which restricts the validity of comparisons and the interpretation of potential differences between populations. Implementing multigroup invariance procedures would have been necessary to establish the robustness of the measurement model across different participant groups.

The study did not report the HTMT (Heterotrait–Monotrait) ratio, which is considered a more sensitive method for evaluating discriminant validity compared to simple inter-factor correlations. The absence of this index limits the ability to detect potential overlaps between latent constructs and may affect the robustness of conclusions regarding factorial discrimination. Future research should incorporate HTMT analyses to strengthen evidence of construct distinctiveness.

Lastly, while the present study offers a thorough psychometric evaluation, it did not examine the scale’s predictive utility in relation to concrete outcomes such as academic performance, peer relationships, or psychological well-being. Establishing such predictive links would further validate the practical relevance of the SIER-C and enhance its utility for practitioners seeking to implement early interventions based on identified emotional and adaptive profiles. Future research should explore these associations, potentially integrating the SIER-C within broader assessment batteries designed to inform intervention strategies across educational and clinical settings.

In sum, while the current study provides compelling evidence for the reliability, validity, and utility of the SIER-C within its targeted demographic, these limitations underscore avenues for future inquiry aimed at consolidating and extending the scale’s applicability. Addressing these limitations will contribute to a more comprehensive and culturally sensitive understanding of emotional intelligence and resilience in childhood, ultimately informing more effective, evidence-based practices in education, psychology, and related fields.

## 5. Conclusions

The SIER-C scale offers a validated and methodologically sound framework for the nuanced assessment of emotional intelligence and resilience in children, addressing a critical need within both research and applied educational contexts. Its robust psychometric properties, demonstrated through rigorous analyses, affirm its capacity to capture complex emotional and adaptive competencies with precision and reliability. Beyond its statistical validity, the scale’s structure allows practitioners to identify specific emotional and resilience-related profiles, thereby facilitating more targeted and individualized intervention strategies.

The practical implications of these findings are significant. The SIER-C can serve as a valuable diagnostic and evaluative tool for psychologists, school counselors, and educational researchers seeking to enhance emotional development and adaptive functioning in children. By providing detailed insights into specific areas of strength and vulnerability, the scale supports evidence-based decision-making in both preventative and remedial educational frameworks. Moreover, the SIER-C aligns with contemporary priorities in educational policy and practice that emphasize the cultivation of social-emotional competencies as foundational to academic success and long-term psychological well-being.

Future research should aim to extend the current findings through longitudinal designs that examine the stability and developmental trajectories of emotional intelligence and resilience across critical stages of childhood and adolescence. Such studies would not only reinforce the scale’s predictive validity but also inform the timing and nature of interventions aimed at optimizing these competencies. Additionally, integrating the SIER-C within broader social-emotional learning (SEL) programs could offer empirical insights into the effectiveness of such interventions, contributing to the refinement of SEL curricula and the development of best practice guidelines.

Ultimately, the SIER-C holds considerable promise as a scientific and practical resource for advancing the understanding and promotion of emotional and adaptive skills in educational settings, supporting the broader aim of fostering resilient, emotionally competent future generations.

## Figures and Tables

**Table 1:** Demographic characteristics of the sample.

Demographic Variable	Category	N	Percentage (%)
Age	6	64	17.4
	7	30	8.2
	8	50	13.6
	9	46	12.5
	10	51	13.9
	11	59	16.1
	12	67	18.3
Class	1	78	21.3
	2	57	15.5
	3	82	22.3
	4	70	19.1
	5	80	21.8
Gender	Male	192	52.3
	Female	175	47.7

**Table 2:** Descriptive statistics for SIER-C Subscales and item-level responses.

	N	Minimum	Maximum	Mean		Std. Deviation	Variance
	Statistic	Statistic	Statistic	Statistic	Std. Error	Statistic	Statistic
S1	367	5	21	9.86	0.181	3.465	12.009
S2	367	9	24	18.72	0.186	3.558	12.659
S3	367	5	23	11.93	0.226	4.330	18.751
S4	367	8	25	20.91	0.238	4.560	20.798
S5	367	9	23	13.83	0.160	3.061	9.368
S6	367	5	25	15.66	0.197	3.774	14.246
I1	367	1	5	1.87	0.049	0.930	0.864
I2	367	1	5	2.05	0.052	0.996	0.992
I3	367	1	5	1.94	0.048	0.911	0.829
I4	367	1	5	1.96	0.049	0.930	0.865
I5	367	1	5	2.03	0.049	0.943	0.890
I6	367	1	5	3.74	0.051	0.976	0.952
I7	367	1	5	3.72	0.052	0.987	0.974
I8	367	1	5	3.74	0.047	0.894	0.799
I9	367	1	5	3.83	0.056	1.067	1.139
I10	367	1	5	3.69	0.044	0.837	0.700
I11	367	1	5	2.35	0.057	1.084	1.175
I12	367	1	5	2.26	0.053	1.018	1.037
I13	367	1	5	2.46	0.056	1.065	1.134
I14	367	1	5	2.45	0.056	1.080	1.166
I15	367	1	5	2.40	0.056	1.066	1.136
I16	367	1	5	4.13	0.063	1.199	1.438
I17	367	1	5	4.18	0.062	1.192	1.421
I18	367	1	5	4.30	0.064	1.225	1.500
I19	367	1	5	4.11	0.063	1.212	1.469
I20	367	1	5	4.19	0.064	1.230	1.514
I21	367	1	5	2.78	0.051	0.968	0.937
I22	367	1	5	2.78	0.050	0.958	0.917
I23	367	1	5	2.79	0.048	0.927	0.860
I24	367	1	5	2.78	0.048	0.917	0.841
I25	367	1	5	2.70	0.049	0.942	0.887
I26	367	1	5	3.17	0.059	1.139	1.297
I27	367	1	5	3.20	0.059	1.124	1.264
I28	367	1	5	3.14	0.060	1.149	1.321
I29	367	1	5	3.07	0.061	1.162	1.350
I30	367	1	5	3.07	0.059	1.132	1.281
Valid N (listwise)	367						

**Table 3:** KMO and Bartlett’s Test Results.

Analysis	Results
KMO Measure of Sampling Adequacy	0.773
Bartlett’s Test of Sphericity	χ^2^ = 3709.485, df = 435, *p* < 0.001

**Table 4:** A. Component Extraction and Explained Variance. B. Rotated Component Matrix (Promax, Loadings > 0.50).

A						
**Component**	**Initial Eigenvalue**	**% Variance**		**Cumulative %**	**Variance After Rotation (%)**	
**1**	**5.221**	**17.40%**		**17.40%**	**11.12%**	
**2**	**3.028**	**10.10%**		**27.50%**	**9.71%**	
**3**	**2.635**	**8.78%**		**36.28%**	**9.52%**	
**4**	**2.152**	**7.18%**		**43.46%**	**9.43%**	
**5**	**1.871**	**6.24%**		**49.69%**	**7.61%**	
**6**	**1.474**	**4.91%**		**54.61%**	**7.22%**	
B						
**Item**	**1**	**2**	**3**	**4**	**5**	**6**
**I11**	**0.796**					
**I12**	**0.802**					
**I13**	**0.796**					
**I14**	**0.762**					
**I15**	**0.786**					
**I16**		**0.622**				
**I17**		**0.731**				
**I18**		**0.815**				
**I19**		**0.668**				
**I20**		**0.700**				
**I6**			**0.790**			
**I7**			**0.719**			
**I8**			**0.763**			
**I9**			**0.730**			
**I10**			**0.704**			
**I1**				**0.670**		
**I2**				**0.769**		
**I3**				**0.744**		
**I4**				**0.705**		
**I5**				**0.705**		
**I21**					**0.630**	
**I22**					**0.688**	
**I23**					**0.593**	
**I24**					**0.585**	
**I25**					**0.709**	
**I26**						**0.659**
**I27**						**0.697**
**I28**						**0.642**
**I29**						**0.565**
**I30**						**0.680**

Notes. Extraction method: Principal Axis Factoring (PAF) for common variance. Rotation method: Promax (oblique), allowing factor correlations. Rotation converged in 6 iterations. Only loadings > 0.50 are presented for clarity.

**Table 5:** A. CFA Model Fit Indices. B. Factor Loadings, AVE, R^2^, and Reliability. C. Fornell–Larcker Criterion for Discriminant Validity.

A						
**Indicator**		**Value**	**Reference Threshold**	**Interpretation**		
Chi-square (Scaled)		556.758	*p* < 0.05	Partial misfit; common in large samples		
Degrees of Freedom (df)		390	—	Appropriate model complexity		
Robust CFI		0.916	>0.90	Good model fit		
Robust TLI		0.906	>0.90	Structural consistency confirmed		
Robust RMSEA		0.043	<0.05	Excellent approximation to population		
SRMR		0.057	<0.08	Acceptable standardized residuals		
Number of Parameters		75	—	Justified by sample size		
Sample Size		367	>200	Adequate for CFA		
AIC		29,191.014	—	Used for model comparison		
BIC		29,483.916	—	Penalizes complexity		
B						
**Factor**	**No. of Items**	**Factor Loadings (Std.all)**	**AVE**	**R^2^ of Indicators**	**Cronbach’s Alpha**	**McDonald’s Omega**
F1	5 (I11–I15)	0.762–0.802	0.61	0.45–0.63	0.84	0.87
F2	5 (I16–I20)	0.622–0.815	0.57	0.41–0.59	0.81	0.85
F3	5 (I6–I10)	0.704–0.790	0.59	0.42–0.63	0.82	0.86
F4	5 (I1–I5)	0.670–0.769	0.55	0.40–0.59	0.80	0.84
F5	5 (I21–I25)	0.585–0.709	0.52	0.36–0.57	0.78	0.82
F6	5 (I26–I30)	0.565–0.697	0.54	0.38–0.58	0.79	0.83
C						
**Factor**	**F1**	**F2**	**F3**	**F4**	**F5**	**F6**
F1	**0.78**					
F2	0.32	**0.75**				
F3	−0.41	0.29	**0.77**			
F4	0.21	0.34	0.18	**0.74**		
F5	0.26	−0.22	0.31	0.27	**0.72**	
F6	0.19	0.28	0.24	0.33	0.30	**0.73**

Notes. AVE > 0.50 indicates convergent validity; R^2^ shows variance explained by latent factors; McDonald’s omega recommended for heterogeneous loadings; factor loadings all significant (*p* < 0.001). Diagonal values = √AVE; off-diagonal = inter-factor correlations; Fornell–Larcker criterion satisfied, confirming discriminant validity.

**Table 6:** Spearman correlations.

			S1	S2	S3	S4	S5	S6
*ρ*	S1	Correlation Coefficient	1.000	−0.051	0.158 **	−0.314 **	0.009	−0.095
		Sig. (2-tailed)		0.329	0.002	0.000	0.870	0.069
		N	367	367	367	367	367	367
	S2	Correlation Coefficient	−0.051	1.000	0.012	−0.059	−0.123 *	0.002
		Sig. (2-tailed)	0.329		0.821	0.256	0.018	0.976
		N	367	367	367	367	367	367
	S3	Correlation Coefficient	0.158 **	0.012	1.000	−0.455 **	0.143 **	−0.113 *
		Sig. (2-tailed)	0.002	0.821		0.000	0.006	0.030
		N	367	367	367	367	367	367
	S4	Correlation Coefficient	−0.314 **	−0.059	−0.455 **	1.000	−0.130 *	0.307 **
		Sig. (2-tailed)	0.000	0.256	0.000		0.012	0.000
		N	367	367	367	367	367	367
	S5	Correlation Coefficient	0.009	−0.123 *	0.143 **	−0.130 *	1.000	−0.136 **
		Sig. (2-tailed)	0.870	0.018	0.006	0.012		0.009
		N	367	367	367	367	367	367
	S6	Correlation Coefficient	−0.095	0.002	−0.113 *	0.307 **	−0.136 **	1.000
		Sig. (2-tailed)	0.069	0.976	0.030	0.000	0.009	
		N	367	367	367	367	367	367

**. Correlation is significant at the 0.01 level (2-tailed). *. Correlation is significant at the 0.05 level (2-tailed).

## Data Availability

The data presented in this study are available on request from the corresponding author.

## References

[B1-ejihpe-16-00008] Al-Elaimat A., Adheisat M., Alomyan H. (2020). The relationship between parenting styles and emotional intelligence of kindergarten children. Early Child Development and Care.

[B2-ejihpe-16-00008] Apostol O. A., Morcov M. V., Sporea C., Morcov M., Morcov C. G., Cioca I. E. (2023). Findings regarding the relationship between parenting styles and sociodemographic parameters in families having children with cerebral palsy. Balneo and PRM Research Journal.

[B3-ejihpe-16-00008] Batool S. S., Lewis C. A. (2022). Does positive parenting predict pro-social behavior and friendship quality among adolescents? Emotional intelligence as a mediator. Current Psychology.

[B4-ejihpe-16-00008] Beer J. (2023). Emotional development in childhood: 3 theories explained.

[B5-ejihpe-16-00008] Cao X., Chen J. (2025). The association between emotional intelligence and prosocial behaviors in children and adolescents: A systematic review and meta-analysis. Journal of Youth and Adolescence.

[B6-ejihpe-16-00008] Cerit E., Şimşek N. (2021). A social skills development training programme to improve adolescents’ psychological resilience and emotional intelligence level. Archives of Psychiatric Nursing.

[B7-ejihpe-16-00008] Charkhabi M., Gavrilova M., Tarasova K., Chichinina E., Dutheil F. (2025). The interplay of non-verbal intelligence and cognitive functions in understanding emotions in preschool children. Psychology Research and Behavior Management.

[B8-ejihpe-16-00008] Chen H., Hong Q., Xu J., Liu F., Wen Y., Gu X. (2021). Resilience coping in preschool children: The role of emotional ability, age, and gender. International Journal of Environmental Research and Public Health.

[B9-ejihpe-16-00008] Cioca I. E., Morcov M. V., Sporea C., Apostol O. A., Morcov C.-G., Ghita M., Pellegrini A., Bordea E.-N. (2025). Parenting styles and parental self-efficacy in parents of children with neurological disorders. Balneo and PRM Research Journal.

[B10-ejihpe-16-00008] Cioca I. E., Morcov M. V., Sporea C., Apostol O. A., Pellegrini A., Bordea E.-N. (2024). Exploring the links between coping strategies, emotional intelligence, and age in adolescents with neuromotor disabilities. Children.

[B11-ejihpe-16-00008] Collaborative for Academic, Social, and Emotional Learning (CASEL) (n.d.). Fundamentals of SEL—CASEL.

[B12-ejihpe-16-00008] Collado-Soler R., Trigueros R., Aguilar-Parra J. M., Navarro N. (2023). Emotional intelligence and resilience outcomes in adolescent period, is knowledge really strength?. Psychology Research and Behavior Management.

[B13-ejihpe-16-00008] Cristóvão A. M., Candeias A. A., Verdasca J. L. (2020). Development of socio-emotional and creative skills in primary education: Teachers’ perceptions about the gulbenkian XXI school learning communities project. Frontiers in Education.

[B14-ejihpe-16-00008] Davis S. K., Wigelsworth M. (2018). Structural and predictive properties of the emotional quotient inventory youth version–Short form (EQ-i:YV[S]). Journal of Personality Assessment.

[B15-ejihpe-16-00008] Dąbkowska M., Kobierecka-Dziamska A., Prusaczyk M. (2021). Anxiety, coping strategies and resilience among children and adolescents during COVID-19 pandemic: A systematic review. Anxiety, uncertainty, and resilience during the pandemic period—Anthropological and psychological perspectives.

[B16-ejihpe-16-00008] Decety J., Holvoet C. (2021). The emergence of empathy: A developmental neuroscience perspective. Developmental Review.

[B17-ejihpe-16-00008] Decety J., Norman G. J., Berntson G. G., Cacioppo J. T. (2012). A neurobehavioral evolutionary perspective on the mechanisms underlying empathy. Progress in Neurobiology.

[B18-ejihpe-16-00008] Denham S. A., Blair K. A., DeMulder E., Levitas J., Sawyer K., Auerbach–Major S., Queenan P. (2003). Preschool emotional competence: Pathway to social competence?. Child Development.

[B19-ejihpe-16-00008] Drigas A., Papoutsi C. (2020). The need for emotional intelligence training education in critical and stressful situations: The case of COVID-19. International Journal of Recent Contributions from Engineering, Science & IT (IJES).

[B20-ejihpe-16-00008] Duckworth A. L., Peterson C., Matthews M. D., Kelly D. R. (2007). Grit: Perseverance and passion for long-term goals. Journal of Personality and Social Psychology.

[B21-ejihpe-16-00008] Fiori M., Agnoli S., Davis S. K. (2023). Editorial: New trends in emotional intelligence: Conceptualization, understanding, and assessment. Frontiers in Psychology.

[B22-ejihpe-16-00008] Fiorilli C., Farina E., Buonomo I., Costa S., Romano L., Larcan R., Petrides K. V. (2020). Trait emotional intelligence and school burnout: The mediating role of resilience and academic anxiety in high school. International Journal of Environmental Research and Public Health.

[B23-ejihpe-16-00008] Goleman D. (n.d.). Creierul şi inteligenţa emoţională: Noi perspective.

[B24-ejihpe-16-00008] Goleman D. (1998). Working with emotional intelligence.

[B25-ejihpe-16-00008] Gordeeva T. O., Osin E. N., Suchkov D. D., Ivanova T. Y., Sychev O. A., Bobrov V. V. (2017). Self-control as a personal resource: Determining its relationships to success, perseverance, and well-being. Russian Education & Society.

[B26-ejihpe-16-00008] Grzesiak E. H. (2024). The importance for a child of emotional intelligence in achieving educational success. Family Upbringing.

[B27-ejihpe-16-00008] Herrera L., Al-Lal M., Mohamed L. (2020). Academic achievement, self-concept, personality and emotional intelligence in primary education. Analysis by gender and cultural group. Frontiers in Psychology.

[B28-ejihpe-16-00008] Hosokawa R., Matsumoto Y., Nishida C., Funato K., Mitani A. (2024). Enhancing social-emotional skills in early childhood: Intervention study on the effectiveness of social and emotional learning. BMC Psychology.

[B29-ejihpe-16-00008] INSSE (2025). Populația rezidentă la 1 ianuarie 2025.

[B30-ejihpe-16-00008] Izard C. E., Trentacosta C. J. (2020). Emotional development. Enciclopedia britannica.

[B31-ejihpe-16-00008] Jefferies P., McGarrigle L., Ungar M. (2019). The CYRM-R: A rasch-validated revision of the child and youth resilience measure. Journal of Evidence-Based Social Work.

[B32-ejihpe-16-00008] Kokkinos C. M., Vlavianou E. (2021). The moderating role of emotional intelligence in the association between parenting practices and academic achievement among adolescents. Current Psychology.

[B33-ejihpe-16-00008] Lopes P. N., Brackett M. A., Nezlek J. B., Schütz A., Sellin I., Salovey P. (2004). Emotional intelligence and social interaction. Personality and Social Psychology Bulletin.

[B34-ejihpe-16-00008] Malik F., Marwaha R. (2018). Developmental stages of social emotional development in children. StatPearls.

[B35-ejihpe-16-00008] Masten A. S. (2001). Ordinary magic: Resilience processes in development. American Psychologist.

[B36-ejihpe-16-00008] Masten A. S. (2014). Global perspectives on resilience in children and youth. Child Development.

[B37-ejihpe-16-00008] Masten A. S. (2018). Resilience theory and research on children and families: Past, present, and promise. Journal of Family Theory & Review.

[B38-ejihpe-16-00008] Maurya S., Suryavanshi P. (n.d.). A review on impact of parenting style on emotional intelligence among college going student. International Journal of Reviews and Research in Social Sciences.

[B39-ejihpe-16-00008] Mavroveli S., Petrides K. V., Sangareau Y., Furnham A. (2009). Exploring the relationships between trait emotional intelligence and objective socio-emotional outcomes in childhood. British Journal of Educational Psychology.

[B40-ejihpe-16-00008] Mayer J. D., Caruso D. R., Salovey P. (2016). The ability model of emotional intelligence: Principles and updates. Emotion Review.

[B41-ejihpe-16-00008] Mayer J. D., Salovey P., Caruso D. (2000). Models of emotional intelligence. Handbook of intelligence.

[B42-ejihpe-16-00008] Mayer J. D., Salovey P., Caruso D. R. (2014). Mayer-Salovey-Caruso emotional intelligence test: Youth research version manual (MSCEIT: YRV manual).

[B43-ejihpe-16-00008] McLaughlin K. A., Garrad M. C., Somerville L. H. (2015). What develops during emotional development? A component process approach to identifying sources of psychopathology risk in adolescence. Dialogues in Clinical Neuroscience.

[B44-ejihpe-16-00008] Metaj-Macula A. (2017). Emotional intelligence, its relation with social interaction and perceived social support. 12th International Conference on Social Sciences.

[B45-ejihpe-16-00008] Musa I. (2024). The power of emotional intelligence in modern education.

[B46-ejihpe-16-00008] Naglieri J. A., LeBuffe P. A., Ross K. M. (2013). Measuring resilience in children: From theory to practice. Handbook of resilience in children.

[B47-ejihpe-16-00008] Nanjundeswaraswamy T. S., Divakar S. (2021). Determination of sample size and sampling methods in applied research. Proceedings on Engineering Sciences.

[B48-ejihpe-16-00008] Paavola L. E. (2017). The importance of emotional intelligence in early childhood.

[B49-ejihpe-16-00008] Prince-Embury S., Prince-Embury S., Saklofske D. H. (2013). Translating resilience theory for assessment and application with children, adolescents, and adults: Conceptual issues BT—Resilience in children, adolescents, and adults: Translating research into practice.

[B50-ejihpe-16-00008] Rutter M. (2012). Resilience as a dynamic concept. Development and Psychopathology.

[B51-ejihpe-16-00008] Saarni C., Camras L. A. (2022). Emotional development in childhood.

[B52-ejihpe-16-00008] Salavera C., Usán P., Quilez-Robres A. (2022). Exploring the effect of parental styles on social skills: The mediating role of affects. International Journal of Environmental Research and Public Health.

[B53-ejihpe-16-00008] Sætren S. S., Sütterlin S., Lugo R. G., Prince-Embury S., Makransky G. (2019). A multilevel investigation of resiliency scales for children and adolescents: The relationships between self-perceived emotion regulation, vagally mediated heart rate variability, and personal factors associated with resilience. Frontiers in Psychology.

[B54-ejihpe-16-00008] Srivastava K. (2013). Emotional intelligence and organizational effectiveness. Industrial Psychiatry Journal.

[B55-ejihpe-16-00008] Sun S., Yan Z., Sun C. (2025). Kindergarten teachers’ emotional intelligence and surface acting: The chain mediating effects of self-efficacy and work engagement. Frontiers in Psychology.

[B56-ejihpe-16-00008] The Modern School (2023). The crucial role of emotional intelligence in education.

[B57-ejihpe-16-00008] Thomas C., Zolkoski S. (2020). Preventing stress among undergraduate learners: The importance of emotional intelligence, resilience, and emotion regulation. Frontiers in Education.

[B58-ejihpe-16-00008] Thompson N. M., van Reekum C. M., Chakrabarti B. (2022). Cognitive and affective empathy relate differentially to emotion regulation. Affective Science.

[B59-ejihpe-16-00008] Weissberg R. P., Durlak J. A., Domitrovich C. E., Gullotta T. P. (2015). Social and emotional learning: Past, present, and future. Handbook of social and emotional learning: Research and practice.

[B60-ejihpe-16-00008] Zeidner M., Matthews G., Roberts R. D., MacCann C. (2003). Development of emotional intelligence: Towards a multi-level investment model. Human Development.

[B61-ejihpe-16-00008] Zhao J.-L., Cai D., Yang C.-Y., Shields J., Xu Z.-N., Wang C.-Y. (2020). trait emotional intelligence and young adolescents’ positive and negative affect: The mediating roles of personal resilience, social support, and prosocial behavior. Child & Youth Care Forum.

[B62-ejihpe-16-00008] Zolkoski S. M., Bullock L. M. (2012). Resilience in children and youth: A review. Children and Youth Services Review.

